# Progress in Medicine

**Published:** 1898-01-29

**Authors:** 


### PROGRESS IN MEDICINE.

### FEVERS.

(*Continued from p* 257.)

**General Pathology.** — Loewy and Richter[1] endeavoured to support the view that pyrexia in itself is not the chief danger. By puncture of the corpus striatum they produced a temperature of 41 deg. C., and then inoculated the animals with the microbes of various fevers. They found the trephined ones lived longer and recovered under larger doses than the other animals, though they had to sustain the additional strain of the operation. The most marked result was obtained in the case of pneumonia. Hence the little value of anti-pyretic drugs except (*a*) those like quinine, which lower the temperature as a result only of their action on the cause of the disease; (*b*) cold baths, which produce elimination and leucocytosis; or (*c*) which are used for extreme hyperpyrexia. It would be possible to treat some fevers by drugs calculated to cause pyrexia if we had any reliable ones available.

Tuohy[2] records an extraordinary hyperpyrexia during typhoid in a nurse. On the thirteenth day she showed a temperature of 112 deg. for four hours, and repeated this performance many times before recovery. On four occasions she reached 117 deg., and on two of these the instrument burst, so that the real point was still higher. The cold pack was employed regularly, but no special investigation into the reality of these temperatures, except the inspection of a brother surgeon, seems to have been made.

Hale White's[3] lectures on the temperature of the body have largely in view the demonstration of fallacies common to many lines of research and the imperfections of calorimetry. He shows, too, that pyrexia is not produced in the same way in all fevers. Turkish baths raise the temperature at first by preventing a loss of heat; puncture of the corpus striatum does so by increased thermogenesis; typhoid probably by the two methods combined; pneumonia chiefly by increased thermogenesis. Erysipelas acts much in the same way, while in pyrexia from suppuration there seems to be a loss of radiation. In many fevers he is able to show that the chemical source of the pyrexia is metabolism of the proteids of the body, and he adds that the administration of grape sugar appears to shield these tissues and lessen the wasting which would otherwise occur.

**Immunity.**—Fraser[4] has investigated the cause of the harmlessness of serpent's poison when taken by the mouth. It is probably not absorbed by the stomach, but is destroyed in the intestine, since it at any rate produces no effect there. When he examined the effect of the bile upon it he found it had considerable power as an antidote, that of venomous serpents being the most active, though that of harmless snakes and oxen was also efficacious. He succeeded in separating the active elements of the bile by precipitation with alcohol, and obtained a substance which could be injected into the blood without harm, and these neutralised a lethal dose of venom. He hopes in this way to obtain an effective natural anti-toxin, and believes that one of the functions of ordinary bile is to neutralise various toxins present in the bowel. In a later paper[5] he further shows that injections of diphtheria toxin mixed with rabbit's bile could be administered to rabbits without harm, while if the bile were omitted death was caused.

Poehl[6] notices that infection must depend in part on the capability of the organism to receive it, and this in turn on the respiration of the tissues. Defects in this function may be caused by (1) low alkalinity of the blood, (2) deficient oxygen, (3) retention of effete products, (4) intestinal fermentation. The last may be inferred when the organic sulphates of the urine are in excess of one-tenth of the organic, or when the indican is much increased. Deficient alkalinity is shown when the bisodic phosphate is less than half the normal. Defective oxidation is present when the urea nitrogen is less than nine-tenths of the total nitrogen excreted, or when the sodium chloride is less than half the urea. Thus, the examination of the urine will disclose many of the conditions which render an animal susceptible to infection. Blumreich and Jacoby[7] extirpated the spleen in a large number of animals, and found that they survived lethal doses of various bacilli; probably through the increased leucocytosis which it produced leading to an increased destruction of bacilli. Calmette[8] shows that immunity may exist, though there is no formation of anti-toxins; thus, the fowl, which is immune against tetanus, affords a serum which has no action on tetanus toxin; that is, contains no substance which destroys it. The preventive power may be a physical and not a chemical phenomenon. The antithermic action of the phenols when painted on the skin has been repeatedly noticed in these columns, and is confirmed by the researches of Gioffredi,[9] who employed various members of the group besides guiacol. He found they appeared in the urine as sulphuric ethers, and caused a fall of temperature by a local, as opposed to a general, action, which brought about an increased loss of heat and a lessened production of it. Attention has also of late been directed to the use of saline injections in various infectious disorders to promote the elimination of the poison; and Dalamere and Descazals[10] give an exhaustive account of the work that has been done on the subject. The injections are followed by a critical reaction with rigors, quickened and difficult breathing; next a hot stage, with flushed face, a rapid strong pulse; and, finally, a fall of temperature with profuse diuresis, diarrhœa, and increased excretion of urea. Injections are made into the veins, such as those of the arm, or the internal saphenous at the ankle; or into the sub-cellular tissue of the flank, axilla, or buttock. The latter are the more painful, and more than one puncture may be needed, but pain may be lessened by adding glycerine or carbolic to the saline injected. In all cases, but more especially in intravenous injections, careful antisepsis should be maintained, and the rate of injection must be always slow. When hæmorrhages have taken place it is found that this treatment tends to prevent a recurrence by favouring coagulability. Various formulæ have been adopted for the liquid used, based on that containing six parts per 1,000 of sodium chloride. Sterilisation is important, and the temperature of the injection should be 104° Fah., to allow of loss in passing through the tubes. Sahli and Bosc have treated typhoid in this way. Dalché reports a cure of septic endocarditis, and Tuffier two cases of tetanus. Where the kidneys are functionally inactive, as in some types of Bright's disease, simultaneous bleeding is used by Barré, but this does not apply to every case of nephritis in an infectious disease.

**Tetanus.**—Höfling[11] treated a case with anti-toxin. Pain began in the neck seven days after a wound in the hand. Eight days later the anti-toxin was injected in one dose of five grains, and was followed by improvement. After a second dose complete recovery took place. Tizzoni[12] had a patient, a pregnant woman, in whom symptoms developed on the eighth day, and in whom the serum was injected on the eleventh and following days for a month, with complete success. In another case the appearance of symptoms was very much slower, and at first treatment by chloral and morphia was employed, followed by anti-toxin. As in most of these slowly-developing cases, the patient gradually recovered. E. J. Smyth[13] reports an instance where infection took place through an old ulcer of the leg. Such a complication is very rare, as the trouble usually affects fresh incised or lacerated wounds. The incubation period was unknown, and a slow recovery followed the use of 11 doses of anti-toxin. Nocard[14] holds that when once symptoms of tetanus have appeared, the anti-toxin cannot prevent a fatal issue. It only cures those who would have recovered without it, but it is nevertheless useful, for it shortens the attack and renders the crises less frequent and severe. If injected after a wound has been received, it either prevents or modifies the attack, according as it has been used at an early or later stage. Donitz,[15] after various tests, comes to the conclusion that the tetanus poison combines with the tissues from the time it enters the blood; that the anti-tetanic serum can liberate and neutralise a fatal dose which has entered the blood not more than eight minutes previously, but that the longer the time the greater the difficulty of separation of the poison from the tissues. He also shows incidentally that the poison may at times produce fatal results without any of the ordinary tetanic symptoms, but merely a slow wasting. Similar results have been observed in diphtheria. Poli[16] gives an instance of the success of the carbolic acid treatment. The symptoms developed five days after the injury. Injections of 1 per cent. carbolic solution were given every two hours, and for a time hourly, until nearly 500 injections had been administered without ill effects. Lambert[17] lays stress on the fact that the bacilli remain localised and not disseminated in the body, and almost always enter when in their spore stage. Unless there is a mixed infection of certain saprophytes at the same time, the tetanus organism cannot develop; indeed, direct infection of one animal from the pus of another dies out after two or three stages. The poison, according to the latest researches, is non-albuminous, and is the most deadly known, since it is 20 times as fatal as the same weight of cobra venom, and 250 times as lethal as strychnine or prussic acid. Courmont endeavoured to prove that the so-called poison is a ferment which forms the true toxin at the expense of the organism, and points to the long period which elapses after its injection before symptoms appear; but most recent observations are opposed to this view. Carbolic and perchloride solutions are of little value for disinfecting the wound, unless they have hydrochloric acid (half per cent) added to them. Iodine trichloride or Gram's solution are efficacious, as are also caustic soda or potash of ·4 per cent. strength. The author has collected 114 cases treated with anti-toxin, and the mortality appears to have been 40·3 per cent. Of the acute cases—that is those where the incubation period was not more than eight days, or if longer followed by extremely rapid onset of symptoms—he finds 47 recorded with 12 recoveries. If we exclude cases which died before the remedy could act, and other doubtful instances, he reckons the recoveries amount to nearly 38 per cent., and the mortality to 61. As the rate under ordinary methods amounts to only 20 per cent. of recoveries, he argues that the remedy is of a certain value, and saves some 18 per cent. of the acute cases. Though the anti-toxin cannot undo the injury already done, it renders inert the toxin circulating in the blood, and any more which may be produced from the focus near the wound afterwards. If possible, preventative injections should, he thinks, be employed whenever there is any reason to dread the occurrence of the disease; but beyond this we can employ local disinfection of the wound, sedatives, and the anti-toxin to annul further action of the poison.

**Rabies.**—Considerable interest was caused by the discussion of a case of acute ascending paralysis which occurred as a complication of Pasteur's treatment,[18] and which resembled the toxic ascending myelitis produced by the injection of certain poisons. The patient pricked his finger at the post-mortem of a person dead from rabies, and underwent 16 injections to prevent infection. Paraplegia, lumbar pain, hyperæsthesia, and rise of temperature followed. The upper limbs next became involved, and the heart beats were much quickened; but the injections were stopped, and recovery quickly took place. It is denied that it could be a form of paralytic rabies from the symptoms and incubation period, and Brouardel himself confesses that the source of the poison is not known, though as an illustration of the rare and usually fatal disease, acute ascending paralysis, the attack was of much interest.

[1] B.M.J., Ap. 24. [2] Lancet, June 26. [3] Lancet, Aug. 14. [4] B. Med. J., July 17. [5] B. Med. J., Sept. 4. [6] B. Med. J., June 5. [7] B. Med. J., Sept. 4. [8] B. Med. J., July 24. [9] B. Med. J., June 26. [10] Med. Chron., July. [11] B. Med. J., July 1. [12] Dublin J. Med. Sc., July 1. [13] Lancet, Dec. 18. [14] Amer. J. M. Sc., Dec. [15] B. Med. J., July 24. [16] B. Med. J., June 12. [17] N. York Med. J., June 5. [18] B. Med. J., July 10.

